# A Self-Referencing Detection of Microorganisms Using Surface Enhanced Raman Scattering Nanoprobes in a Test-in-a-Tube Platform

**DOI:** 10.3390/bios3030312

**Published:** 2013-09-13

**Authors:** Nan Xiao, Chao Wang, Chenxu Yu

**Affiliations:** Department of Agricultural and Biosystems Engineering, Iowa State University, Ames, IA 50011, USA; E-Mails: nxiao@iastate.edu (N.X.); chaow@iastate.edu (C.W.)

**Keywords:** surface enhanced Raman scattering, self-referencing, microorganism, nanoparticles

## Abstract

Anisotropic nanoparticles (*i.e*., silver nanocubes) were functionalized with target-specific antibodies and Raman active tags to serve as nanoprobes for the rapid detection of bacteria in a test-in-a-tube platform. A self-referencing scheme was developed and implemented in which surface enhanced Raman spectroscopic (SERS) signatures of the targets were observed superimposed with the SERS signals of the Raman tags. The assessment through the dual signals (superimposed target and tag Raman signatures) supported a specific recognition of the targets in a single step with no washing/separation needed to a sensitivity of 10^2^ CFU/mL, even in the presence of non-target bacteria at a 10 times higher concentration. The self-referencing protocol implemented with a portable Raman spectrometer potentially can become an easy-to-use, field-deployable spectroscopic sensor for onsite detection of pathogenic microorganisms.

## 1. Introduction

The presence of pathogenic bacteria in food and drinking water poses a threat to both public health and security; approximately 9.4 million illnesses, 56,000 hospitalizations and 1,300 deaths occur annually in the U.S. [1]. To deal with this threat, an early-warning surveillance system is needed to detect bacteria at the earliest possible moment, preferably in-field. However, traditional biochemical and nutritional analysis for microorganisms, such as analytical profile index (API), requires a series of biochemical tests for cultured microbes that takes days to finish [2]. A molecular assay, such as sandwiched Enzyme-linked immunosorbent assay (ELISA), cuts the experimental time to hours; however, in ELISA assay, multiple washing steps are needed to separate bound antibodies from unbound ones, which still limits their in-field deployability [3]. Surface enhanced Raman scattering (SERS)-based signaling is emerging as an alternative to fluorescence-based detection [4,5]. In recent years, the advances in SERS-based biosensing have enabled detection of targets at even single molecule sensitivity [6,7,8]. SERS-based molecular probes typically contain nanoscale metallic structures such as the SERS enhancer, organic Raman tags as quantitative reporters for the presence of the probes, and capturing molecules (*i.e*., antibodies, single strand DNAs, RNA aptamers) to interact with bio-targets [9,10,11,12]. Their application bears great similarity to fluorescence-labeled antibodies, with the advantage of broader reporter library (theoretically, any molecule with a specific spectrum can be used as a Raman reporter, hence the restriction on selecting a Raman reporter is quite limited), supreme multiplexing due to sharp Raman bands (~10 nm *vs*. fluorescence band of ~50–300 nm) [4,5], and no photobleaching. A detection limit of 10 cells/mL has been reported by Wang and coworkers with the SERS-active labeling technique [12]. However, to utilize the SERS-probes for target detection, separation of target-bound probes from unbound ones through multiple washing steps is still needed, which reduces its field deployability.

An alternative to SERS probe-based detection is to directly identify bio-targets using their own inherent Raman fingerprints. Utilizing self-assembled monolayers of metallic nanoparticles, well-controlled nanostructures can be fabricated to yield reproducible SERS substrates for biosensor applications [11,12,13,14,15,16,17,18,19,20]. Kahraman *et al*. reported a limit of detection (LOD) of 10^3^ CFU/mL for *E. coli* in water [13]. With SERS and a novel barcode data processing procedure, Ziegler *et al*. reported more than 10 bacteria could be differentiated [14]. However, direct differentiation/identification of microbial targets through their unique Raman spectroscopic signatures requires high-quality and highly reproducible spectral data in conjunction with statistical analysis built upon known spectral fingerprints of the microbial species. Well-defined and controlled nano-SERS substrates used in these spectroscopic sensors usually only provide moderate enhancement [6], and highly-sensitive benchtop Raman spectrometers are needed to produce the required high-quality spectral data, which limits the field depolyability of these approaches. Besides, SERS decays exponentially away from the surface of the nanostructures, in nanoprobes where surfaced-immobilized antibodies (or other capturing molecules) are used, biotargets are usually not in direct contact with the metallic surface, and the enhancement to the biotarget Raman spectral signals is therefore much weaker, and further increases the difficulty to acquire high-quality SERS spectra directly from the bio-targets. 

Another concern over SERS-based microorganism detection and identification is that SERS signals from microorganisms are highly dependent on the types of the SERS substrates [15,16,17], how SERS substrate are brought into contact with the microbial targets [18,19,20], and the excitation laser wavelengths [18,19,20]. Variations in these factors may lead to a lack of consistency and poor reproducibility across measurements, which limit the application of SERS-based detection. 

**Figure 1:**
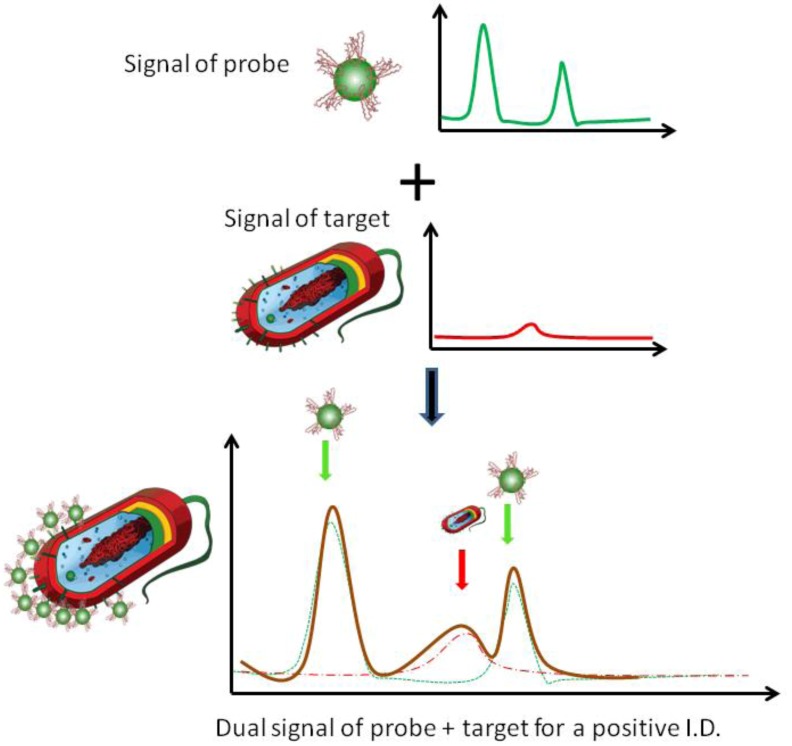
Schematic illustration of a dual recognition mechanism for surface enhanced Raman spectroscopic (SERS) pathogen detection; the superimposed bacterial signature peaks and probe signatures peaks indicate the binding between the bacterial target and the nanoprobes.

In this study we introduced a concept of self-referencing mechanism that utilizes SERS molecular probes to achieve bacteria detection in one single step in a test-in-a-tube platform, which would be more suitable for in-field applications. As shown in Figure 1, in the dual-recognition mechanism, Raman-labeled and antibody-functionalized anisotropic nanostructures (e.g., silver nanocubes) are fabricated as SERS nanoprobes that display specific probe signatures (probe signal), and through covalently-bound antibodies they could bind to their target bacterial cells specifically. The selection of silver nanocube as the SERS probe is to take advantage of the superior electromagnetic enhancement of anisotropic nanoparticle to Raman scattering of target molecules in their vicinity, due to the geometrical singularity of these nanoparticles [21,22,23,24]. The antibody-antigen binding ensures that the target cells would bind to enough nanoprobes, and measureable SERS signals from the bacterial cells would be generated (non-target would NOT have enough nanoprobes bound to them, and their SERS signal would be non-measurable due to weak or no enhancement). Observation of superimposed SERS signals of the probe and the target indicates the binding events, and subsequently definitely identifies the target in one single step; no washing or separation is needed. Furthermore, since the specificity of the target detection is provided by the antibodies, only a few key signature peaks from the bacterial cells are needed for the target recognition, a positive identification of the target can be reached without relying on multivariate analysis throughout a broad spectral range. A portable Raman sensor thus becomes feasible under the self-referencing dual-recognition scheme for target detection, as shown in this study. 

For this self-referencing scheme to work properly, the selection of proper Raman tags to label the nanoprobes is critical. The scheme relies on the observation of Raman peaks from the microbial cells to confirm binding between the nanoprobes and the microbes. Since microbial cells are further away from the nanoparticle surface, enhancement to microbial Raman signal is not as strong as to the Raman tags. Hence, if the peaks from the Raman tags are in the same spectral range as the peaks from the microbes (primarily cell walls), the weaker microbial signal may be overwhelmed by the stronger Raman tag signal, and fail to provide the self-referencing confirmation of the binding events. In an earlier report [25], we used 11-mercaptoundecaonic acid (MUDA) as the Raman tag. The strong peaks of MUDA overlap with that of microbial cells to a great extent; only two weak microbial peaks were identified for the self-reference. In this study, a different Raman tag (4-aminothiophenol, 4-ATP) was used, which only has weak signals below 900 cm^−1^. Microbial Raman peaks below 900 cm^−1^ hence can be easily recognized for self-reference.

## 2. Experimental Section

### 2.1. Reagents and Antibodies

Ethylene glycol (99%), sodium sulfide (Na_2_S, 99%), Polyvinylpyrrolidone (PVP, 99%), silver nitrate (>99%), 4-aminothiophenol (4-ATP, >99%), sodium nitrite (>99%), sodium hydroxyl (>99%) were all purchased from Sigma-Aldrich (St. Louis, MO, USA) and used without further purification. Nanopure deionized and distilled water (18.2 MΩ) was used for all experiments. 

Polyclonal anti-*E. coli* (Pierce^®^) antibodies (Lot# LC1293681) were purchased from Thermo Scientific (Rockford, IL, USA) for *E. coli* molecular detection. *E. coli* and *L. monocytogenes* cultures were provided by a colleague (Brehm-Stecher). 

### 2.2. Nanoprobe Fabrication and Functionalization

**Figure 2:**
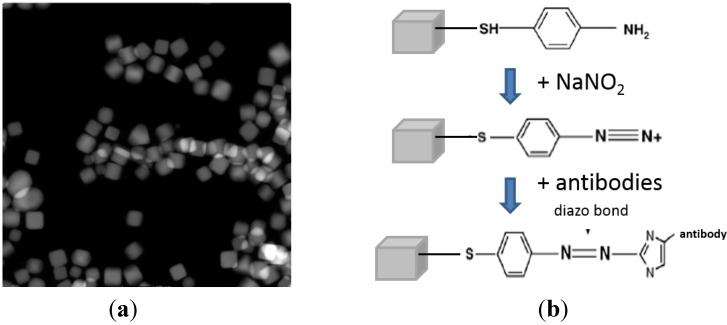
(**a**) Silver nanocubes used to make molecular probes (STEM image); (**b**) Reaction scheme to functionalize silver nanocubes with anti-*E. coli* antibodies.

To take advantage of the superior surface enhancement performance of anisotropic nanoparticles due to the stronger local electromagnetic field around the singular features (sharp corners and edges) of these particles [6,20], we chose to use Ag nanocubes (as shown in Figure 2(a)) to make nanoprobes. 

Silver nanocubes were synthesized following the method of Skrabalak *et al.* [26]. Typically, in 20 mL glass reaction vial, 6 mL of ethylene glycol were added. The vial was incubated at 150 °C for 1 h to evaporate the water in the system. After heating, 70 μL of 3 mM Na_2_S in ethylene glycol was pipetted to each vial. The vial was then heated for 10 min before 1.5 mL of 0.02 g/mL PVP (Polyvinylpyrrolidone) in ethylene glycol was added together with 0.5 mL of 0.048 g/mL AgNO_3_ in ethylene glycol. Finally, the whole mixture was heated for up to 20 min until the solution became ochre-colored.

The reaction was quenched by putting the vial in water bath at room temperature. The nanocubes were subsequently rinsed with acetone and then washed with nanopure water for three times and finally resuspended in 4 mL of nanopure water.

The PVP coating of the Ag cubes was then replaced with 4-ATP for antibody attachment. The 4-ATP molecules also serve as Raman tag for the nanoprobes. To replace the PVP coating with 4-ATP, 4 mL of 3 nM Ag-PVP were reacted with 0.5 mL of 10 mM 4-ATP dissolved in acidic water (pH = 2) under vigorously stirring at 60 °C for 3 h. The solution was then centrifuged and washed with water-free ethanol once and acidic water (pH = 4) twice to remove the extra PVP and unbound 4-ATP. Finally, Ag-4-ATP nanocubes were redispersed in 2.5 mL of acidic water (pH = 4).

To covalently conjugate antibodies with the Ag-4-ATP nanocubes, diazonium salt of Ag-4-ATP was obtained by reacting 1 mL of as made Ag-4-ATP solution with 10 mL of 10^–3^ M sodium nitrite at pH 4 at 0 °C for 30 min [9]. Then the Ag-4-ATP-azo solution was neutralized by addition of 300 µL 1 M sodium hydroxyl and 1 mL of 100 mM phosphate buffer (pH = 7.4). Finally, antibodies were incubated with Ag-4-ATP solution at 0 °C for 4 h to form diazo bonds between the electron-rich aromatic lateral chains of antibody molecules and the diazonium moiety of the Ag-4-ATP-azo [9], the unbound antibodies were successively removed by centrifugation, and the Ag-4-ATP-Abs were collected and resuspended in 10 mM phosphate buffer (pH = 7.4) and stored at 4 °C. The functionalization reaction scheme is illustrated in Figure 2(b). The final concentration of the nanoprobe was ~5 nM. They remain stable for up to 1 month. 

### 2.3. Bacterial Cell Culture

Two bacterial strains (*E. coli* and *L. monocytogenes*) were grown in LB medium at 37 °C for 18 h. The bacterial cells were then centrifuged and washed with PBS buffer twice and finally redispersed in PBS buffer. The final bacterial cell concentration was determined by optical density (OD) measurement at 600 nm (the concentration of the bacterial cells for both strains was ~10^9^ CFU/mL at OD = 1.0) 1 mL of the PBS solution with various cell concentrations was then incubated with proper nanoprobes over ice for around 30 min in an eppendorf tube (1.5 mL) to allow the nanoprobes to bind to their targets, respectively. 

### 2.4. One-Step Raman Spectroscopic Measurement

**Figure 3:**
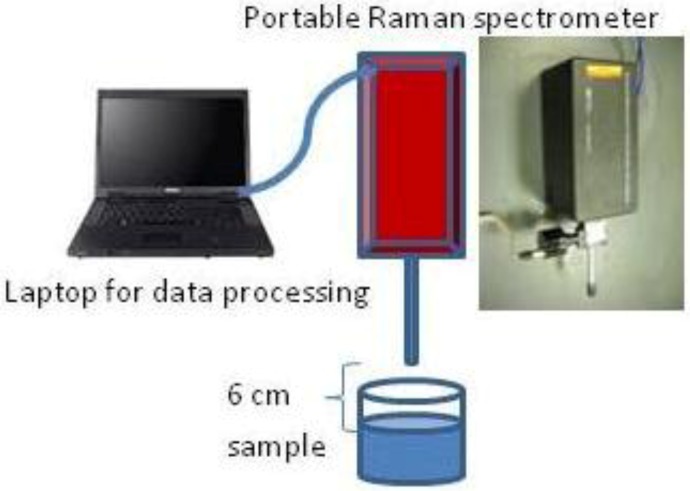
Schematic illustration of the experimental setup.

To acquire SERS spectra of the bacteria-nanoprobe sample, 10 µL of the sample was withdrawn from the eppendorf tube and put into a cap of an eppendorf tube (removed from a tube). The cap was used as a sampling container. It was put at the focal point (6 cm measuring from the tip of the *i-*Raman spectrometer (B&W Tek, Inc., Newark, DE, USA) as shown in Figure 3) of the *i*-Raman laser beam (15 mW, 785 nm NIR laser, 3 cm^−1^ spectral resolution, 400–2,000 cm^−1^ range). The focal point of the laser beam was pre-set by the manufacturer. Raman spectra of the sample were hence obtained directly with no washing being performed after the mixing of the bacterial suspension and the nanoprobes in one single step. 60 s integration time was used at 15 mW laser power for spectral acquisition. 10 spectra were collected from each sample to calculate an average spectrum, which was used for further analysis. 

### 2.5. Spectral Data Processing

Using near infrared excitation (785 nm) radically reduces the observance of sample auto-fluorescence. To further reduce the remaining fluorescence, a polynomial background subtraction method was implemented [27,28]. Another challenge in spectral pre-processing is to capture important patterns in the spectra while removing noise or other fine-scale structures. The 10-point moving average method was used in this study to smooth the spectra. Finally all spectra were area-normalized for intensity consistency at the region between 400 cm^−1^ to 2,000 cm^−1^. All data processing was conducted using R, a widely used language and software tool for statistical computing and graphics. Peak intensity is calculated based on the integration of the peak area.

## 3. Results and Discussion

### 3.1. Self-Referencing Detection of Target Bacteria in a Test-in-a-Tube Platform

The diazo-conjugation strategy utilizes aromatic side chains of the antibodies for their immobilization onto the Ag-cubes [29,30,31,32,33]. As the side chains that are reactive can be at the Fc segment as well as the Fab segment of the antibody molecule, the orientation of the immobilized antibody molecules on the Ag-cube surface is uncertain. In certain cases the antigen binding sites might be brought in close vicinity to the Ag-cube surface; when such antibody molecules bind to their target cells, the cell walls are also being brought into close distance to the Ag-cube surface, and SERS signals can be observed from these cells. 

**Figure 4:**
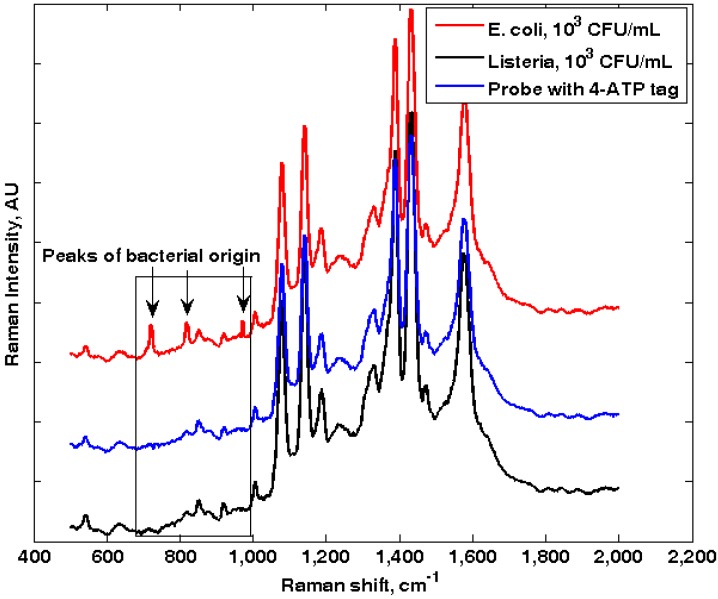
SERS spectra of silver nanoprobes functionalized with anti-*E. coli* antibodies (blue) incubated with *E. coli* (red) and *Listeria* (black). No bacterial-originated peaks can be observed when non-target species is integrated with the nanoprobes.

Figure 4 shows the Raman spectra of Ag-4-ATP-Abs probe (Ag-nanocube functionalized with anti-*E. coli* antibodies) binding with *E. coli* and *L. monocytogenes*, respectively. Since bacteria cells were suspended in aqueous solution, they are constantly drifting in and out of the focal volume of the laser beam. To assure a meaningful SERS spectrum is acquired, the integration time was set to be 60 s, with the laser power set at 15 mW. Earlier reports suggested that at this power level and integration time, microbial samples tend to become graphitic and their spectroscopic characteristics may change dramatically [20]. However, in our experiment the cells were suspended in aqueous solution, and the SPR-induced heat damage seems to be alleviated. Also the cells were drifting in and out of the focal volume, and the actual laser exposure of individual cells seems not to have gone over the graphitic limit; evidenced by the lack of the D and G bands of graphene in the spectra. 

Peaks at 735 cm^–1^ and 1,330 cm^–1^ have been widely reported as characteristic SERS peaks that can be observed from microbial cells [15,16,17,18,19,20]. Due to the nature of surface enhancement, only molecules that are in close vicinity to the SERS substrate can be affected by the surface-plasmon based enhancement. The 735 cm^–1^ and 1,330 cm^–1^ peaks are typical of adenine [20]. They can come from the adenine part of the fully reduced FAD [34], or from other adenine-bearing molecules (NAD, ATP, DNA, *etc*.). No matter what molecular species that contributes to the adenine peaks, they can only come from the cells. The 735 cm^–1^ peak only appeared upon the binding of nanoprobes to their specific bacterial targets (*E. coli*). For samples with only probes or with a non-target bacteria *L. monocytogenes*, the 735 cm^–1^ peak is missing. Hence, this peak was used subsequently as the fingerprinting peak for *E. coli* in the self-referencing mechanism. 

It has been reported that the 1,330 cm^–1^ peak in bacteria is usually stronger than the 735 cm^–1^ peak, hence should be a better indicator of probe-bacteria binding. However, 4-ATP itself displays a strong peak at ~1,335 cm^–1^, which completely overwhelms the “could-be” bacterial peak at the same wavelength region. As shown in Figure 4, a peak is always visible at ~1,330 cm^–1^, regardless of samples. Hence, in our experiment the 1,330 cm^-1^ adenine peak cannot be used as a bacterial marker.

Two other peaks (825 cm^–1^ and 985 cm^–1^) also appeared to be associated with the presence of target bacteria (*E. coli*) in a sample, also their exact biological origins are less clear and more investigation is needed. In a summary, as illustrated in Figure 1, the observation of bacteria-originated Raman peaks in the superimposed probe-target spectrum clearly indicated the presence of the targets in a sample. The absence of bacteria-originated peaks in control samples as well as non-target samples showed that the self-referencing detection scheme is highly specific and accurate. 

Our observation was consistent with what has been previously reported [20]: Although colloidal nanoparticles can be absorbed onto microbial cell wall from all directions due to non-specific electrostatic interaction, simply mixing bacterial cells with nanoparticles usually do not bring enough particles to the surface of bacterial cells to generate measurable SERS signals. With our specifically functionalized nanoprobes, however, significantly more nanoprobes were attached to the surface of the target cells due to the antibody-antigen binding, and in certain cases the molecules located at the inner side of the cell walls come into close proximity to the Ag-cubes that are bound on the surface, and the 735 cm^–1^ peak becomes detectable. 

Due to the usual longer distance between the cellular originated molecular species (*i.e*., FAD, NAD *etc*.) and the nanoparticle surface (*i.e*., no direct contact) than that between the Raman tag (4-ATP molecules) and the nanoparticle surface (direct contact), the enhancement to the Raman tag is more effective and the observed spectra were dominated by the signatures of the 4-ATP molecules. However, the dual-recognition mechanism only requires a few identifiable signature peaks from the microbial cells to serve as spectral markers, as demonstrated in Figure 4. 

### 3.2. Limit of Detection of the Dual-Recognition Probing Scheme

The limit of detection (LOD) and specificity of the self-referencing detection scheme were investigated. A detectable signal is recorded when the peak intensity measured from a sample is significantly higher (≥M_control_ + 3σ) than the peak intensity of a control, where M_control_ is the mean of the peak intensity of the control (10 replicates), and σ is the standard deviation of the peak intensity of the control at the same wave number.

**Figure 5:**
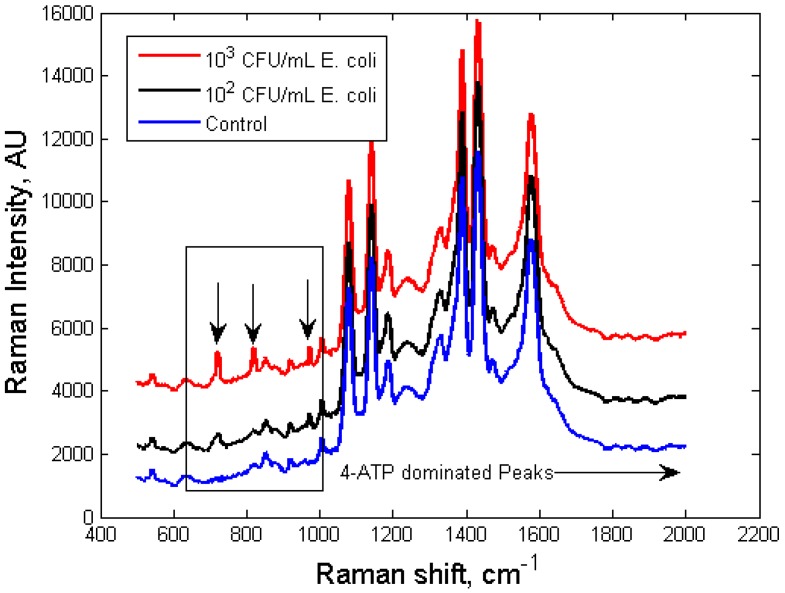
SERS spectra of silver nanoprobes functionalized with anti-*E. coli* antibodies, incubated with *E. coli* (red and black) at different concentrations (blue: control sample with no *E. coli* cells).

**Figure 6:**
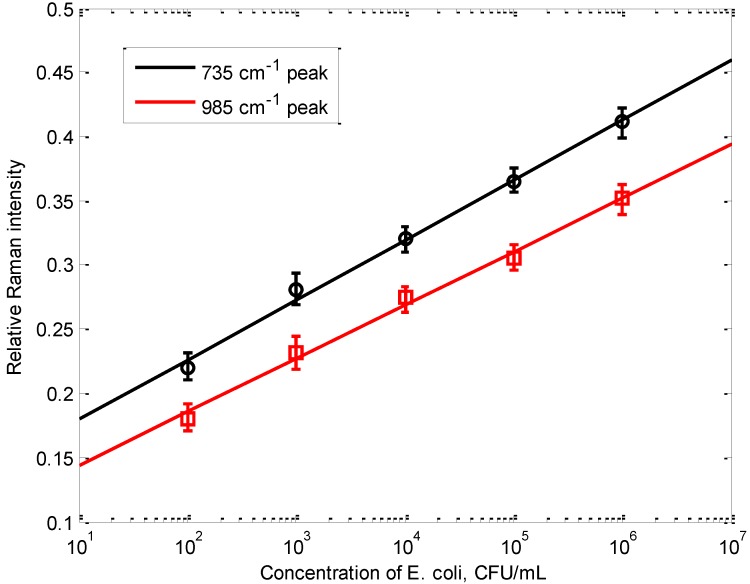
The correlation between the relative Raman intensity of the 735 cm^–1^ peak and the concentration of *E. coli* cells in the sample. The relative Raman intensity was calculated using the 4-ATP peak at 1,079 cm^–1^ as internal reference, error bar indicates the standard errors of the mean from five independent runs.

As shown in Figure 5, the 735 cm^–1^ bacterial peak became detectable at 10^2^ CFU/mL *E. coli* concentration, comparable to that of high-end ELISA assay, without going through any washing steps. When *E. coli* concentration was lower than 10^2^ CFU/mL (three were tested, 50 CFU/mL, 30 CFU/mL, 10 CFU/mL, respectively), the 735 cm^−1^ peak was not significantly different from that of the control (within M_control_ ± 3σ), hence it was considered as indistinguishable from the control. The relative intensity of the 735 cm^–1^ peak, calculated with reference to the 1,079 cm^–1^ peak (representative peak of 4-ATP, which is determined by the number of nanoprobes, not the number of bacterial cells, stayed within M_control_ ± 3σ for all samples investigated), is exponentially correlated to the concentration of bacterial cells in the sample, between 10^2^ CFU/mL to 10^6^ CFU/mL. A second peak at 985 cm^−1^ was also investigated. Similarly, the relative intensity of the 985 cm^−1^ peak calculated with reference to the 1,079 cm^−1^ peak, is exponentially correlated to the concentration of bacterial cells in the sample. Experiments were replicated five times with each concentration, and the standard errors of the mean for each concentration are also shown in Figure 6. The self-referencing assay potentially can be developed into a fast semi-quantitative assay for bacterial targets. 

To further improve the LOD of the self-referencing scheme, more effective SERS enhancers should be used. The Ag nanocubes are good SERS enhancers, due to their sharp corners and edges that generate singularity in electromagnetic field around the particles which enhances the Raman scattering electronically. Nano-enhances with more extreme singularity, such as nanostars or nano-prisms, may provide stronger enhancement that would result in better LOD. 

### 3.3. Specificity of the Dual-Recognition Probing Scheme

**Figure 7:**
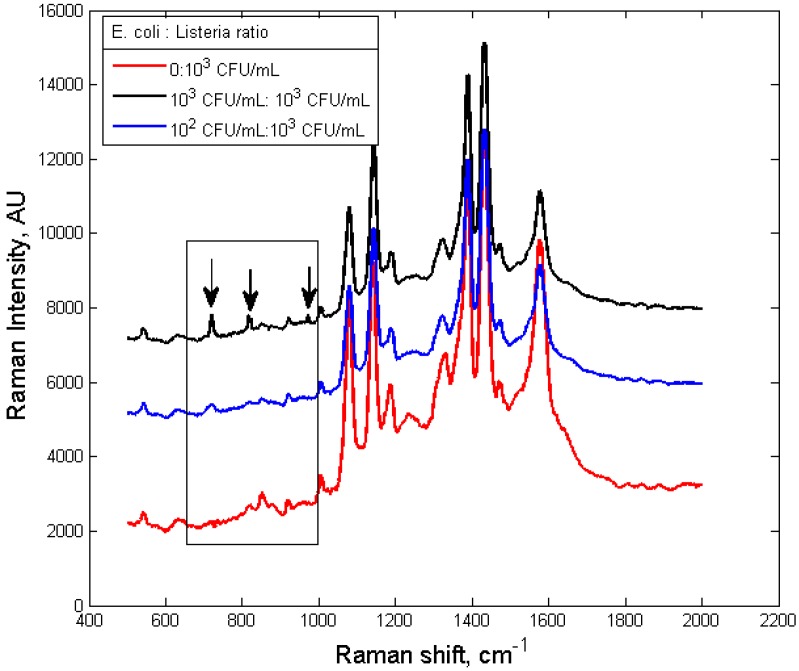
SERS spectra of silver nanoprobes with anti-*E. coli* antibodies interacted with *E. coli* and *Listeria* mixture sample solutions with different *E. coli:Listeria* ratio.

The specificity of the self-referencing dual-recognition scheme is determined by the specificity of the antibodies. As illustrated in Figure 7, when a sample with non-targets (*L. monocytogenes*, 10^3^ CFU/mL) was integrated by the nanoprobes (specifically *E. coli*), no self-referencing signatures (735 cm^–1^, 825 cm^–1^ and 985 cm^–1^) were observed which resulted in a correct negative I.D. For a sample with a 10-fold higher non-targets (*L. monocytogenes*, 10^3^ CFU/mL) concentration than target (*E. coli*, 10^2^ CFU/mL), the specific *E. coli* peaks could still be identified, as shown in Figure 7. Relatively high level of interferences from other bacteria (*L. monocytogenes*) did not diminish the accuracy of the self-referencing dual-recognition scheme, indicating that this scheme would be extremely attractive to in-field pathogen detection applications, where interference from other co-existed microorganism species will be omnipresent. 

## 4. Conclusions

A self-referencing mechanism was successfully developed for single-step detection of bacterial target in a lab-in-a-tube setting using SERS spectroscopic sensing. The detection time, about 30 min, is relatively short compared to alternative methods. The sensitivity of the self-referencing probing scheme is high (10^2^ CFU/mL), and it was demonstrated that interference from other sources was minimal even at high concentrations (10 times higher than the targets). As the binding efficiency between nanoprobes and target bacteria is further improved through the optimization of the nanoprobe design and functionalization, stronger SERS enhancement of the fingerprinting peaks of the bacterial targets could be expected. In addition, by deploying nanoprobes with molecular recognition agents targeting different motifs (e.g., antibodies binding to different epitopes), a multiplexing self-referencing scheme can be further developed to improve the detection specificity. With the self-referencing mechanism, a multiplexing field-deployable biosensor can soon be developed for onsite detection of pathogenic bacteria with high sensitivity and selectivity.

## Figures and Tables

**Figure S1:**
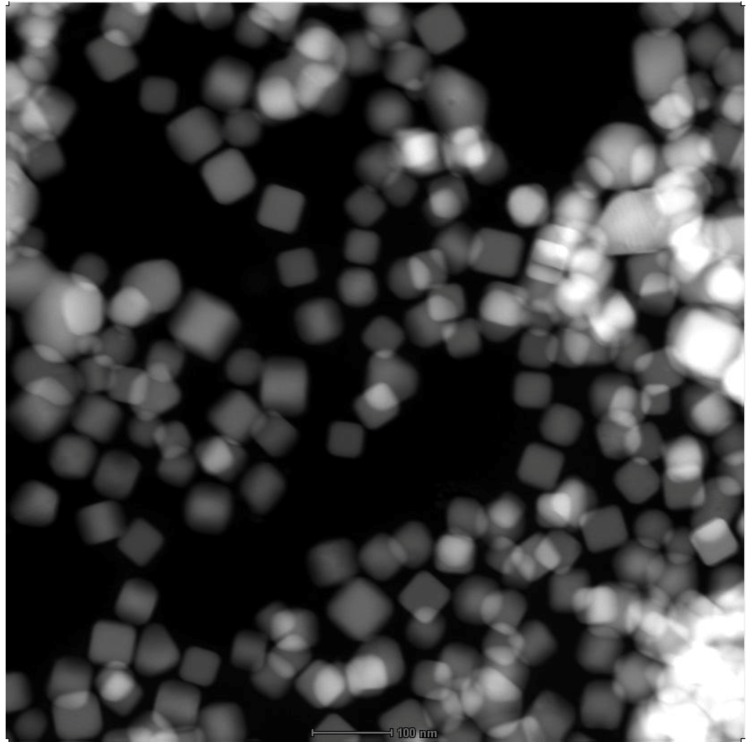
STEM image of the silver nanocube.

**Figure S2:**
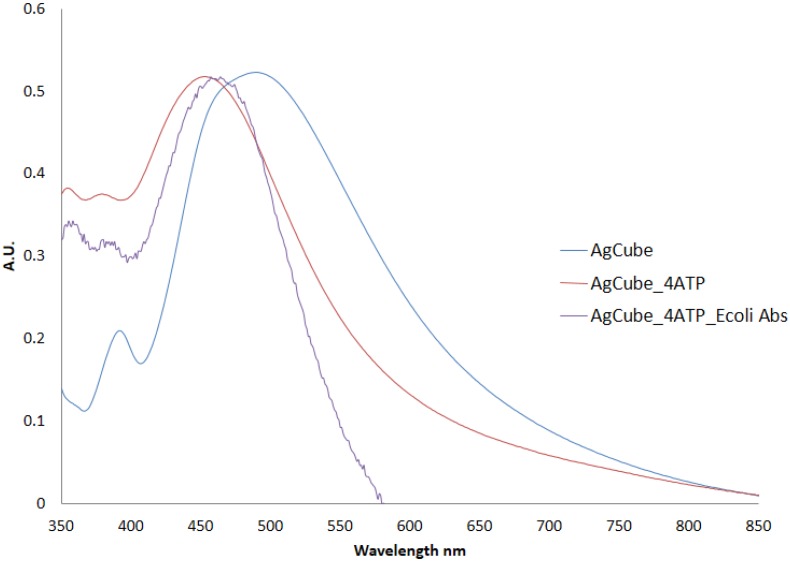
Extinction spectra of of silver nanocube (PVP coated), 4-ATP-coated silver nanocube, and antibody-functionalized silver-nanocube probes.

**Figure S3:**
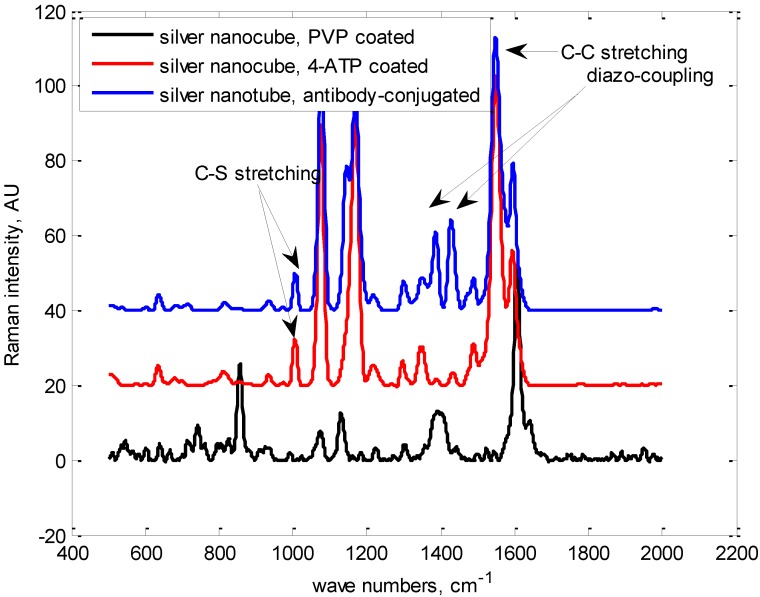
Raman spectra of Ag nanocube with antibodies conjugated to them.

## Appendix

### Synthesis and Bio-Functionalization of Silver Nanocubes

The silver nanocubes synthesized in this study have a average size of 41 nm, with a standard deviation of 7.8 nm, measured over 437 particles imaged using STEM. Typical STEM image is shown in Figure S1.

Extinction spectra of silver nanocube (PVP coated, right after synthesis), 4-ATP-coated silver nanocube, and antibody-functionalized silver-nanocube probes are shown in Figure S2. After replacing the PVP coating with 4-ATP, the plasmonic peak blue-shifted, due to the change of refractive index (a reduction) and the thickness of the surface coating; the thinkness of the 4-ATP coating, which is a monolayer of small molecules, is much smaller than that of the PVP coating. After antibodies are conjugated to the 4-ATP linker, the plasmonic peak re-shifted slightly, due to the addition of the antibody molecules further increased the refractive index of the surface layer on the nanoparticles.

Figure S3 shows the Raman spectra of PVP-coated silver nanocube, 4-ATP-coated silver nanocube and anti-*E. coli* antibody-conjugated silver nanocube. A layer of 4-ATP molecules were anchored on the surface of the silver nanotubes after incubation with the Ag-cube solution due to Ag–S bonding. As shown in Figure S2, band at 1,090 cm^−1^ is the stretching vibration of C–S bond and band at 1,590 cm^−1^ is the C–C stretching vibration of benzene ring in 4-ATP [29,30,31,32,33]. The appearance of these bands, replacing the original PVP bands, indicated the successful replacement of PVP with 4-ATP on the Ag-cube surface. Another notable difference between the spectra of pure 4-ATP and that of 4-ATP labeled Ag-cube is the intensity of 4-ATP characteristic peak at 1,590 cm^−1^. The apparent enhancement of the mode at 1,590 cm^−1^ can be ascribed to a charge transfer between the metal and the 4-ATP molecules [9], further confirms the binding of 4-ATP to the Ag-cube surfaces.

The Ag-4-ATPs were then reacted with nitrite ions in acid condition to form diazonium salt, which subsequently reacted with histidine residues of the antibodies. The stengtherning of the 1,394 cm^−1^ and 1,435 cm^−1^ diazonium peaks (N=N stretching) proved the conjugation of the antibodies, as shown in Figure S3 [9,29,30,31,32,33].
